# ASPP2κ Is Expressed In Human Colorectal Carcinoma And Promotes Chemotherapy Resistance And Tumorigenesis

**DOI:** 10.3389/fmolb.2021.727203

**Published:** 2021-11-05

**Authors:** Ingmar Rieger, Vasileia Tsintari, Mathis Overkamp, Falko Fend, Charles D. Lopez, Marcus M. Schittenhelm, Kerstin M. Kampa-Schittenhelm

**Affiliations:** ^1^ Department of Oncology, Hematology, Clinical Immunology and Rheumatology, University Hospital Tübingen (UKT), Tübingen, Germany; ^2^ Institute of Pathology at the University Hospital Tübingen, Tübingen, Germany; ^3^ Department of Hematology and Medical Oncology, Oregon Health and Science University (OHSU), Portland, OR, United States; ^4^ Clinic of Medical Oncology and Hematology, Cantonal Hospital St. Gallen (KSSG), St. Gallen, Switzerland; ^5^ Translational Experimental Hematology and Oncology, Medical Research Center and Department of Oncology and Hematology, Cantonal Hospital St. Gallen, St. Gallen, Switzerland

**Keywords:** colon cancer, tumorigenesis, therapy resistance, alternative splicing, TP53, ASPP2, ASPP2κ, apoptosis

## Abstract

Alternative splicing is a common physiologic mechanism to generate numerous distinct gene products from one gene locus, which can result in unique gene products with differing important functional outcomes depending on cell context. Aberrant alternative splicing is a hallmark of cancer that can contribute to oncogenesis and aggressiveness of the disease as well as resistance to therapy. However, aberrant splicing might also result in novel targets for cancer therapy. ASPP2 is a haplo-insufficient tumor suppressor, that functions through both p53-dependent as well as p53-independent mechanisms to enhance cell death after stress. Interestingly, the common human tumor *TP53* mutations result in a loss of the binding sites to ASPP2, leading to impaired induction of apoptosis. Vice versa, attenuation of ASPP2 has been described to be associated with high-risk disease, therapy failure and poor clinical outcome especially in tumors harboring the *TP53* wildtype (WT) isoform. We have recently identified a novel, dominant-negative splicing variant of *ASPP2*, named *ASPP2κ*, with oncogenic potential. Exon-skipping results in a reading-frame shift with a premature translation stop, omitting most of the ASPP2 C-terminus - which harbors the p53-binding domain. Consequently, the ASPP2-p53 interaction is abrogated, which in part impacts on oncogenesis, aggressiveness of disease and response to therapy. Since *ASPP2κ* has been shown in hematologic malignancies to promote tumorigenesis, we further wished to determine if aberrant *ASPP2κ* expression plays a role in human solid tumors. *In this report,* we find that ASPP2κ is frequently expressed in human colorectal tumors (CRC). Using ASPP2κ overexpressing and interference CRC models, we demonstrate a functional role of ASPP2κ in contributing to oncogenesis and resistance to therapy in CRC by 1) enhancing proliferation, 2) promoting cell migration and, 3) conferring resistance to chemotherapy induced apoptosis. Our findings have far-reaching consequences for future diagnostic and therapeutic strategies for ASPP2κ expressing colorectal cancer patients and provide proof-of-principle to further explore ASPP2κ as potential predictive marker and target for therapy in clinical trials.

## Introduction

Among all tumors, the incidence of colorectal cancer (CRC) ranks third worldwide in terms of incidence and second in terms of mortality (approx. 1.8 million, resp. 881,000 in total in 2018) (Bray et al., 2018). In the advanced metastatic stage, treatment options are mostly palliative except in selected circumstances (Chen et al., 2021). Moreover, despite increased knowledge and therapeutic options for metastatic CRC (Sveen et al., 2020), there remains a critical need for prognostic as well as predictive biomarkers to direct more effective therapies.

The current understanding of tumorigenesis supports the idea of a stepwise malignant transformation of cells by acquisition of alterations of genes that interfere with cell fate, cell survival, and genome maintenance (summarized by Vogelstein and colleagues (Vogelstein et al., 2013)). In CRC malignant transformation typically has a latency of several decades.

The tumor suppressor protein p53 (*TP53*), also referred to as “the guardian of the genome”, plays a crucial role in the prevention of tumor formation and TP53 related apoptosis pathways are central mechanisms in cellular stress response and tumor suppression (Bergamaschi et al., 2004). Approximately 50% of all colorectal cancers show *TP53* inactivating gene mutations associated with poor clinical outcome (Yin et al., 2018). We and others have shown that dysregulation of the ASPP protein family displays an alternative mechanism to inactivate the p53 pathway, especially in *TP53* wildtype (WT) cells (Vives et al., 2006; Kampa et al., 2009a). Two pro-apoptotic members (ASPP1 and ASPP2) and the anti-apoptotic variant (i)ASPP have been described (Bergamaschi et al., 2004; Trigiante and Lu, 2006; Sullivan and Lu, 2007). These proteins share an evolutionary conserved C-terminus including four-ankyrin repeats, a SH3-domain and a poly-proline-rich domain, which directly interact with the p53 core domain (Iwabuchi et al., 1994; Gorina and Pavletich, 1996; Samuels-Lev et al., 2001; Takahashi et al., 2004; Ahn et al., 2009).

ASPP1 and ASPP2 promote p53-dependent apoptosis (Iwabuchi et al., 1998). In contrast, iASPP binds to an adjacent linker region to inhibit apoptosis (Bergamaschi et al., 2006). Intriguingly, altered expression levels of ASPP1, ASPP2 or iASPP have been observed in CRC (Yin et al., 2018). However, the functional and clinical consequences of ASPP family isoforms have not been explored in CRC.

We now present data showing that the dominant-negative oncogenic splicing variant of *ASPP2* (*ASPP2κ*) (Schittenhelm et al., 2019) is aberrantly expressed in human CRC. We find inhibition of therapy-induced apoptosis as well as an increase in tumor cell proliferation and migration. Our data provides the rationale for further investigations addressing the role of *ASPP2κ* as a potentially clinically useful biomarker and therapeutic target in CRC.

## Materials and Methods

### Cell Lines

Two colorectal cancer cell lines (DLD-1 p53+/+ and HCT116 p53+/+), both a gift from Prof. Vogelstein (John-Hopkins-University, Baltimore, MD) were maintained in McCoy´s 5A (modified) Media (Gibco) supplemented with 10% foetal bovine serum (FBS) (Sigma Aldrich), 1% penicillin-streptomycin (Biochrom), 1% Sodium pyruvate and 1% Non-Essential Amino acids (100X) (Gibco). For lentiviral particle production, HEK293T cells (Thermo Fisher) were maintained in Hyclone- Dulbecco’s Minimum Essential Media (DMEM, Gibco), supplemented with 10% FBS and 200 μM L-glutamine. All cell lines were kept in an incubator at 37°C in 5% CO2.

### Patient Tissue Analysis

Fresh, snap-frozen tissue, collected from 15 consented patients diagnosed with primary CRC (G2—G3) was obtained from the central Biobank of the Comprehensive Cancer Centre Tübingen-Stuttgart. The project was approved by the local ethics committee (188/2018BO2). Tumor-free surrounding tissue from the same patients served as controls.

### RNA Extraction and Real Time-Quantitative Polymerase Chain Reaction

For RNA preparation and qRT-PCR, RNA was extracted using the RNeasy^®^ RNA purification kit (Qiagen). cDNA synthesis was performed using the Reverse Transcriptase Kit (Roche). To determine *ASPP2κ* expression levels, a qRT-PCR assay was established to run with qRT-PCR Roche^®^ LightCycler Technology (Roche) and Light Cycler 480 Probes Master (Roche). 18s served as housekeeping reference gene. All primer sets were purchased from Eurofins and are shown in Table 1. Measurements were performed in triplicates. Relative quantification of the target gene transcript in comparison to the reference transcript was calculated using the Cp method.

**TABLE 1:** Primers used for qRT-PCR.

Primer name	Sequence (5′-3′)
ASPP2κ forward	CTG​CTG​ATA​GTG​ATG​GAT​GGA​G
ASPP2κ reverse	CCC​AAA​GCG​CAT​AAA​TGA​CT
18s forward	ATCCCTGAAAAGTTC
18s reverse	CACACCCTTAATGGC

### ASPP2κ Isoform-Specific Interference

Recombinant lentiviral particles expressing a custom-made 29-mer short hairpin (sh) RNA against *ASPP2κ* were produced and transduced into the CRC cells according to the manufacturer’s protocols. Empty vector (EV) strains served as controls.

Briefly, a pre-selected custom synthesized lentiviral construct was designed (pGFP-C-shLenti vector; Origene) containing a shRNA expression cassette against *ASPP2κ*, driven by an U6 promoter, a puromycin resistance marker, driven by a SV40 promoter and a tGFP, driven by a CMV promoter. HEK293T cells were lipofected (Lipofectamine 2000; Thermo Fisher) for lentiviral particle production following the manufacturer’s protocol (packaging mix, Dharmacon). The lentiviral particles were used to transduce HCT116 or DLD-1 cells to induce stable hairpin expression against *ASPP2κ*. The transduction efficiency was evaluated determining GFP expression, while puromycin was used as a selection marker.

### Enforced ASPP2κ Expression Model

A lipofection approach was followed to create ASPP2κ overexpressing cell lines, using a custom-made HisMax vector (Eurofins) encoding the peptide sequences of the ASPP2κ splicing variant and a Zeocin resistance site. A HisMax LacZ vector served as an empty vector control (Schittenhelm et al., 2019).

## Apoptosis Assay

Cells were seeded 1 day prior to treatment at 40–50% confluency. Oxaliplatin (Ox) was obtained from Sigma-Aldrich and dissolved in H2O. A dose-dilution assay was set up and cells were cultured for 48 h at 37°C. Cells were then harvested and stained for AnnexinV/PI or APC/7AAD according to manufacturer´s guidelines (Life Technologies) and measured on a FACS Calibur (Becton Dickinson). Experiments were performed in triplicates.

### Proliferation Assay

The number of living cells per well was assessed daily using a hemacytometer after trypan blue staining. Experiments were performed in technical triplicates. Cellular doubling times were determined using the following formula:

$$t_{d}=\;\frac{\ln\ln\;\left(2\right)*t\;}{x_{t}-\;x_{0}}$$

t_d_ = doubling time[h]. t = duration[h]. x_0_ = cell number at timepoint 0. x_t_ = final cell number at timepoint t.

### Wound Healing Assay

For the wound healing assay, cells were grown to 100% confluency. A 20 µl pipette tip was used to create a defined scratch wound. Wound closure was monitored by light microscope (Nikon) with 10x magnification every 2 h for a total of 12 h and additionally after 24 h. Cell migration was quantified and compared to the corresponding control cell strains using NIS Elements software (Nikon).

### Transwell Migration Assay

To determine the migration capacity, a transwell migration assay was established: Cells were seeded in 1.5 ml McCoy (Gibco) pure medium into the upper transwell migration inserts with 8 µm pores. 2.5 ml 20% FBS McCoy medium were added to the bottom chamber. After allowing the cells to migrate for 16 h, all cells from the upper insert including the top of the membrane were removed using a cotton tip. The penetrated cells in the bottom chamber, including cells attached to the bottom side of the transwell membrane, were collected, fixed with 70% ethanol, and stained with a Giemsa (Merck KGaA) solution. Experiments were performed in triplicates. For quantitative analysis, a picture was taken from five random fields using a light microscope (Nikon) at a magnification of 20x and the migrated cells were counted (Mohle et al., 2000).

### Statistical Analysis

Statistical analyses were carried out using GraphPad Prism. Quantitative variables were analysed by Student’s paired and/or unpaired *t*-test, and 2way ANOVA as indicated. All statistical analyses were two-sided, and *p* < 0.05 was considered statistically significant.

## Results

### Detection of ASPP2κ in Human CRC

To determine if *ASPP2κ* is expressed in human CRC, an isoform-specific qRT-PCR assay was established to screen for *ASPP2κ* mRNA expression levels in snap-frozen primary tumor tissue obtained from 15 patients with newly diagnosed adenocarcinoma (G2/3). Patient characteristics are provided as Supplementary Table S1. A pool of tumor-free colon tissue (n = 14) was used as a control cohort.

**FIGURE 1:**
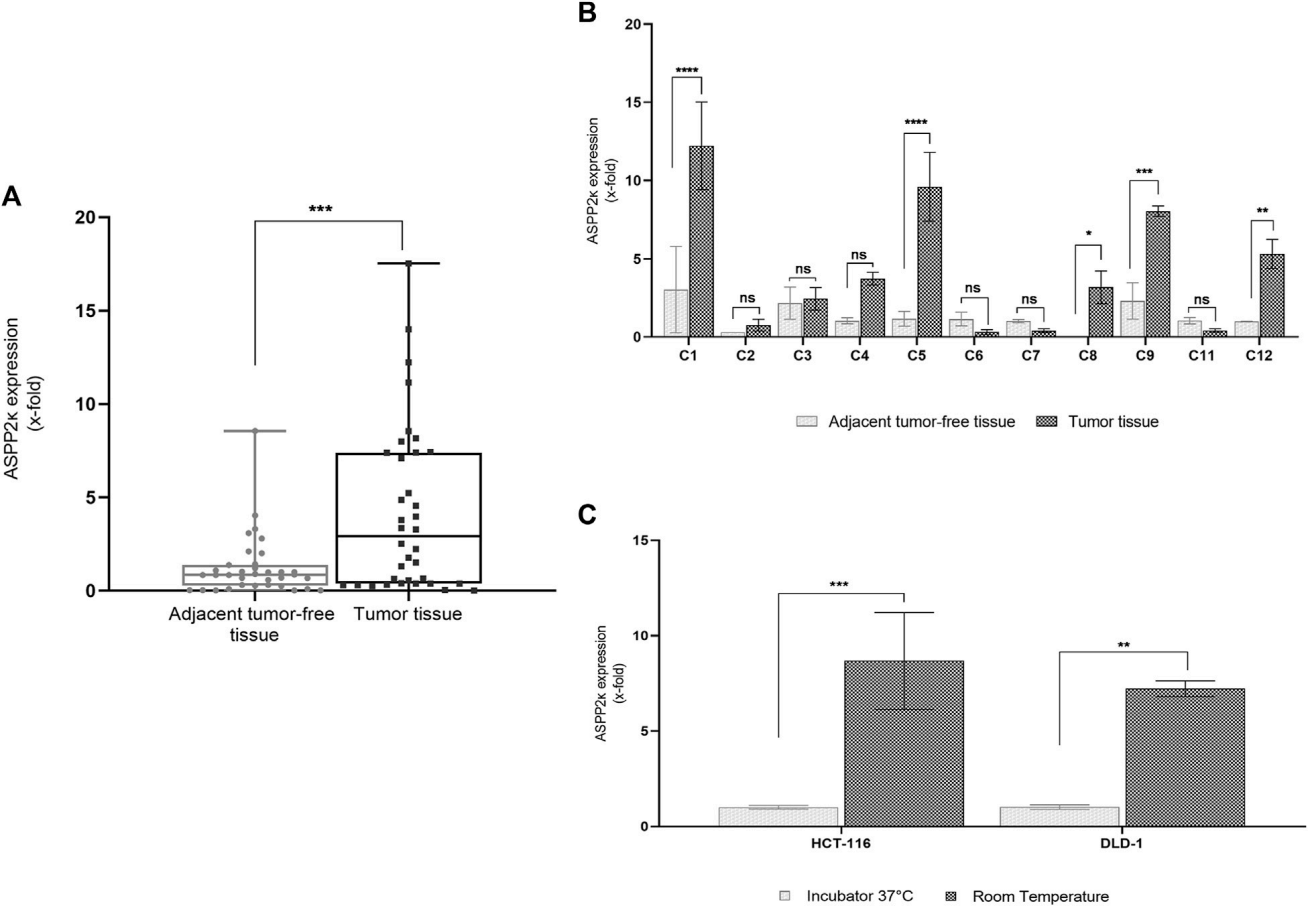
**(A)** ASPP2κ isoform-specific qRT-PCR analysis determines relative mRNA expression levels in CRC tissue (n = 15) compared to a pool of adjacent tumor-free tissue colorectal tissue (n = 14). Analyses performed in triplicates. Statistical tests: unpaired *t*-test **(B)** ASPP2κ specific qRT-PCR assay to compare individual ASPP2κ expression levels in tumor tissue vs. adjacent tumor-free tissue colorectal tissue of the same patient. Statistical test: two-way ANOVA. Pair C10 was removed from analysis due to R1 resection status. **(C)** ASPP2κ specific qRT-PCR demonstrates temperature inducible expression of the splicing variant in CRC cell lines. Statistical test: two-way ANOVA. ****p < 0.0001, ***p < 0.001, **p < 0.01, *p < 0.05, ns: not statistically significant.

We found that on average, *ASPP2κ* is significantly overexpressed in CRC tumor samples compared to normal tissue (*p* < 0.001). Interestingly, expression levels varied widely, ranging from non-expressors to a >10-fold increase in a subset of samples (Figure 1A).

Comparison of individual paired tumor vs. adjacent microscopically tumor-free colon tissue samples (n = 11) revealed significant upregulation of *ASPP2κ* in approx. half of the tested samples. Importantly, expression of *ASPP2κ* was thereby specifically linked to the tumor tissue (Figure 1B).

### ASPP2κ Is Stress-Inducible in CRC

We reported previously that *ASPP2κ* expression is inducible in leukemia (Schittenhelm et al., 2019) in response to different stressors including temperature, UV light or chemotherapy. To explore if *ASPP2κ* expression is stress-inducible in CRC, we utilized CRC human cell lines DLD-1 (Dukes CRC; *TP53* WT (Vives et al., 2006)) and HCT116 (poorly differentiated CRC from primary site; *TP53* WT (Sullivan and Lu, 2007)). Cells were cultured at 37°C or room temperature (RT) overnight and mRNA levels of *ASPP2κ* were quantified in relation to a baseline. In both cell lines, exposure to room temperature stress resulted in a statistically significant induction of *ASPP2κ* expression compared to 37°C (Figure 1C).

### ASPP2κ Expression Modulates CRC Sensitivity to Chemotherapy-Induced Apoptosis

**FIGURE 2:**
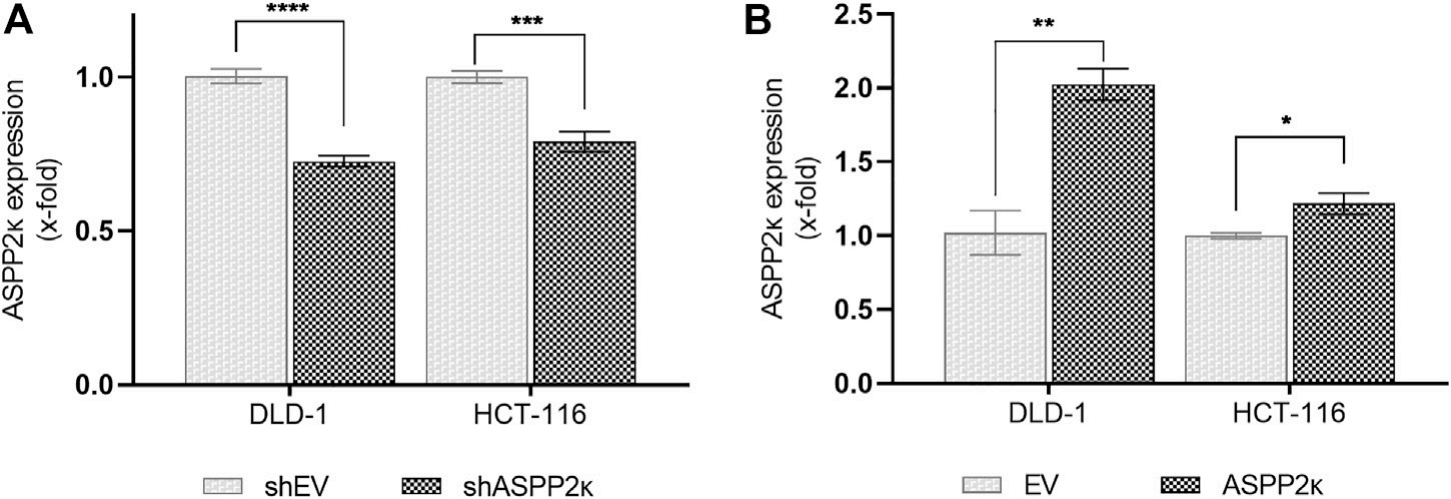
**(A)** Hairpin mediated ASPP2κ-interference or **(B)** forced overexpression of ASPP2κ determined by isoform-specific qRT-PCR. EV, empty vector. Statistical test: two-way ANOVA. ****p < 0.0001, ***p < 0.001, **p < 0.01, *p < 0.05.

In order to assess whether ASPP2κ has an effect on induction of chemotherapy-induced apoptosis, we modified ASPP2κ expression in DLD-1 and HCT116 cell lines, exposed them to the DNA-intercalating agent oxaliplatin (Ox) (a clinically important therapeutic for patients with CRC) and then measured the degree of induction of apoptosis. To silence ASPP2κ, we used an isoform-specific shRNA approach as confirmed by qRT-PCR for each cell line, showing a decrease in *ASPP2κ* expression of 27% for DLD-1 and 21% for HCT116 (Figure 2A). To enforce ASPP2κ expression, the same cell lines were transfected with a HisMax vector encoding for *ASPP2κ* using a lipofection approach. Overexpression of *ASPP2κ* was achieved with a 2-fold increase in the DLD-1 model and a 20% increase of expression levels for the HCT116 cell line (Figure 2B).

**FIGURE 3:**
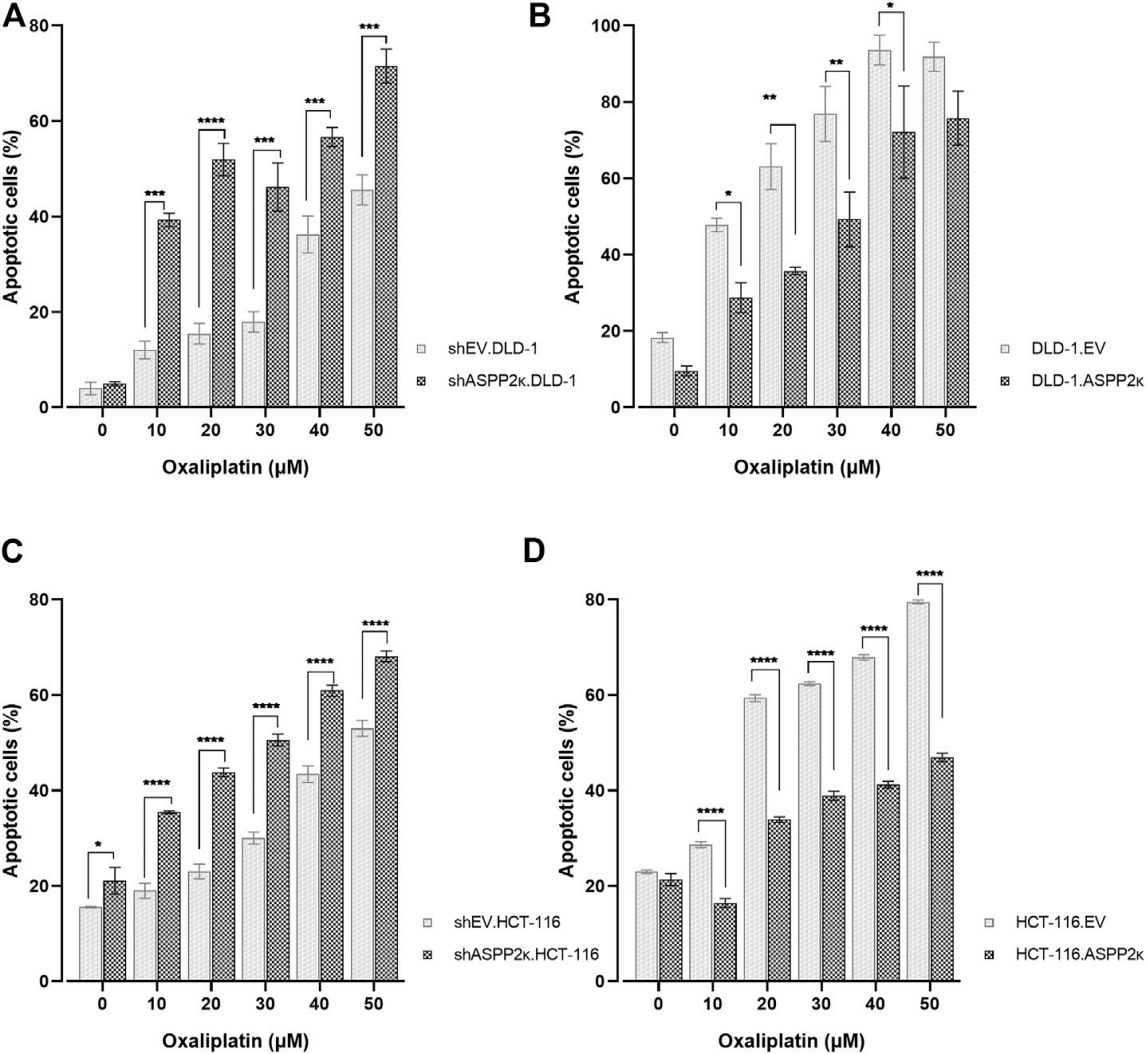
Dose dilution experiments using **(A)** DLD-1 or **(C)** HCT116 ASPP2κ silenced strains **(B)** DLD-1 or **(D)** HCT116 ASPP2κ overexpressing strains are shown. Induction of apoptosis after exposure of cell strains to Ox for 48 h is assessed flow cytometrically using annexin V/7-AAD. EV, empty vector. Statistical test: two-way ANOVA. ****p < 0.0001, ***p < 0.001, **p < 0.01, *p < 0.05.

Given that ASPP2κ promotes resistance to apoptosis in human leukemia, we reasoned that enforced expression in CRC cells would promote resistance to apoptosis. As expected, *ASPP2κ* interferenced cell lines were more sensitive to oxaliplatin-induced apoptosis compared to EV controls (Figures 3A,C). In contrast, cell lines overexpressing *ASPP2κ* were more resistant to apoptosis induction in response to oxaliplatin when compared to the EV control strains (Figures 3B,D). Interestingly, even though the degree of *ASPP2κ* knockdown and overexpression was modest (<2-fold), yet the effect on oxaliplatin-induced apoptosis was significant. This data underscores the important dominant-negative function of the *ASPP2κ* splicing variant (Schittenhelm et al., 2019).

### ASPP2κ Promotes Cellular Proliferation

**FIGURE 4:**
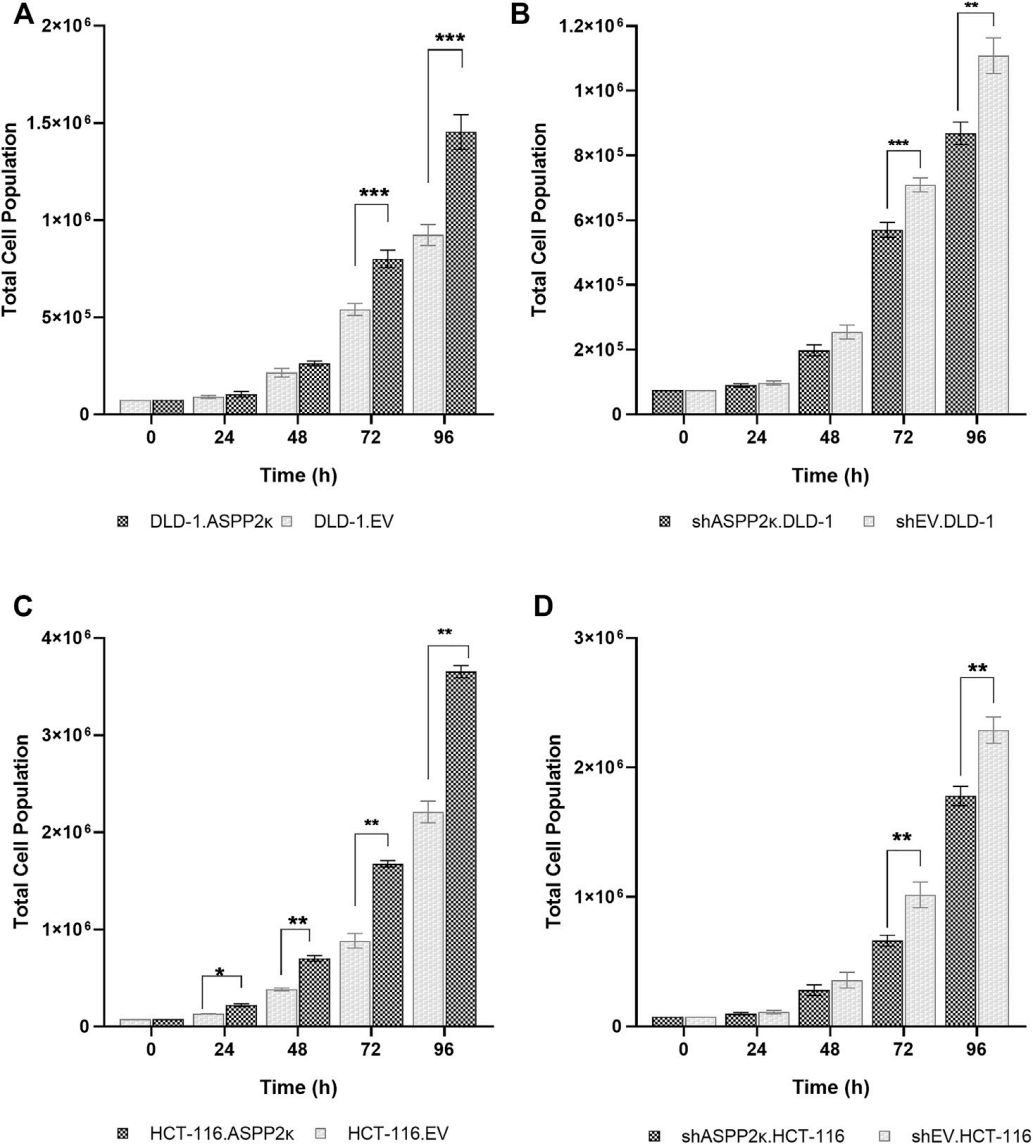
Cell doubling times of CRC cell lines in dependence of ASPP2κ: **(A)** DLD-1 or **(C)** HCT116 strains overexpressing the ASPP2κ isoform or **(B)** DLD-1 or **(D)** HCT116 ASPP2κ silenced strains are shown. EV, empty vector. Statistical test: two-way ANOVA. ***p < 0.001, **p < 0.01, *p < 0.05.

Since ASPP2κ harbors pro-tumorigenic functions (Schittenhelm et al., 2019), we wished to explore how ASPP2κ altered cellular proliferation. We therefore quantified cell doubling times of DLD-1 and HCT116 cell lines modified for ASPP2κ expression. Consistent with a pro-tumorigenic function, forced expression of ASPP2κ results in a significant increase in cellular proliferation rates compared to the empty vector (EV) control cells. Cell doubling times of ASPP2κ enforced expression DLD-1 cells was reduced by 16% from 26.4 h (EV) to 22.3 h leading to a significant increase of proliferation rates at 72 and 96 h (Figure 4A). A similar finding was observed for HCT116 ASPP2κ overexpressing cells with a reduction of cell doubling times by 14% from 19.7 h (EV) to 17 h, which results in a statistically significant increase of proliferation rates over time (Figure 4C). In contrast, the ASPP2κ-attenuated cell strains demonstrated an increase in cell doubling times by approximately 10% from 24.4 h (EV) to 26.9 h for the DLD-1 cell line, and from 19.5 to 21.2 h for the HCT116 strains—both leading to a significant decrease in cellular proliferation rates at 72 and 96 h (Figures 4B,D).

These observations are consistent with the notion that ASPP2κ harbors significant dominant-negative functions that promote CRC tumorigenesis.

### ASPP2κ Enhances Cellular Migration

**FIGURE 5:**
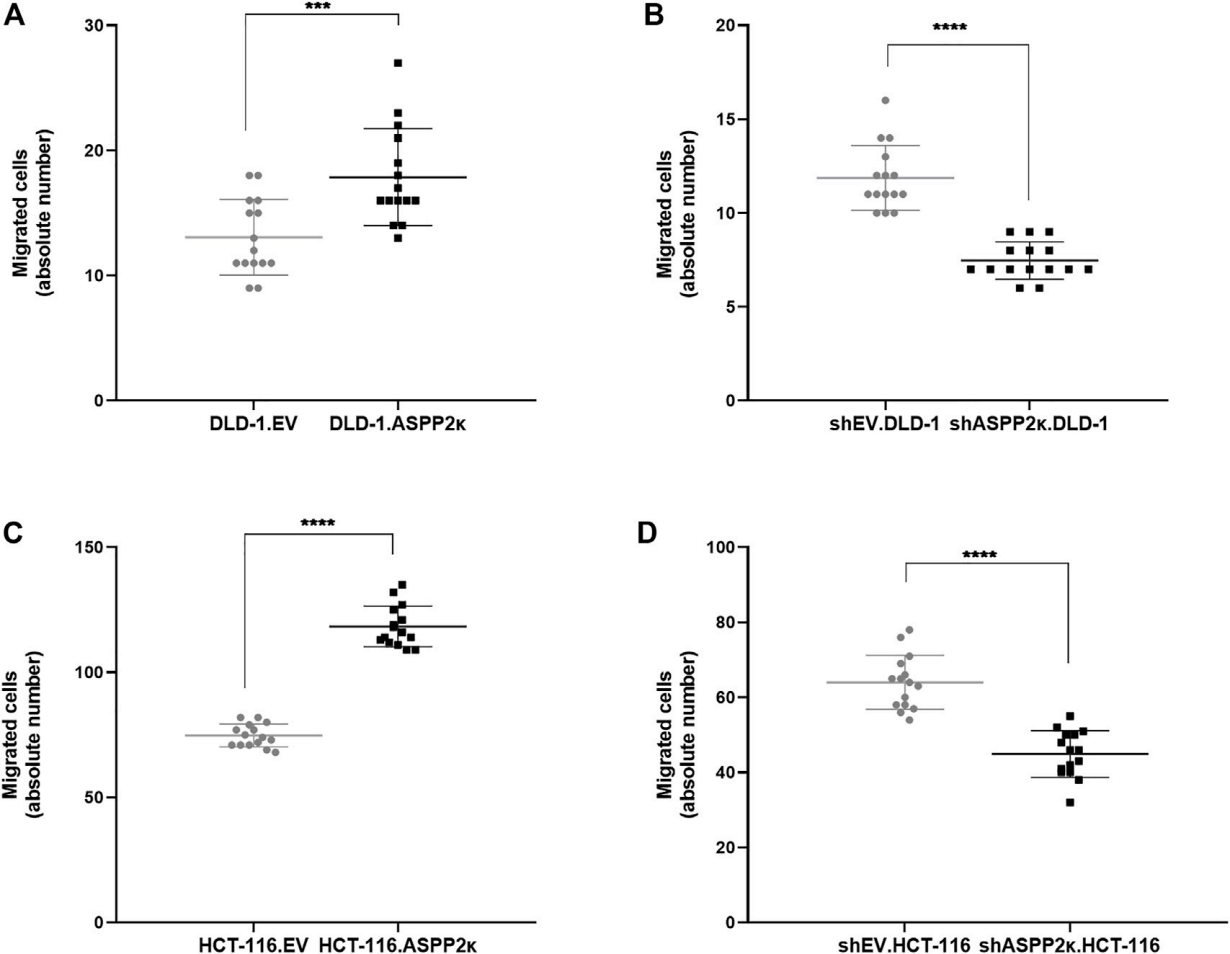
**(A–D)** Quantitative analysis of migrating cells in a transwell migration assay are shown for DLD-1.ASPP2κ **(A)** and HCT-116.ASPP2κ **(C)** cells, resp. the ASPP2κ-interferenced corresponding cell strains for DLD-1 **(B)** and HCT116 **(D)**. Statistical tests: unpaired *t*-test. ****p < 0.0001, ***p < 0.001, **p < 0.01, *p < 0.05.

Since tumor cell migration and invasion are hallmarks of cancer development (Hanahan and Weinberg, 2000), we wished to evaluate how ASPP2κ levels influence the migratory behaviour of CRC cells. Using an established transwell migration assay as previously described (Mohle et al., 2000), we found that forced expression of ASPP2κ resulted in an enhancement of cellular migration with approximately 36 and 58% higher migration rates for DLD-1-ASPP2κ and HCT116-ASPP2κ cell lines when compared to the corresponding EV control cell lines (Figures 5A,C).

**FIGURE 6:**
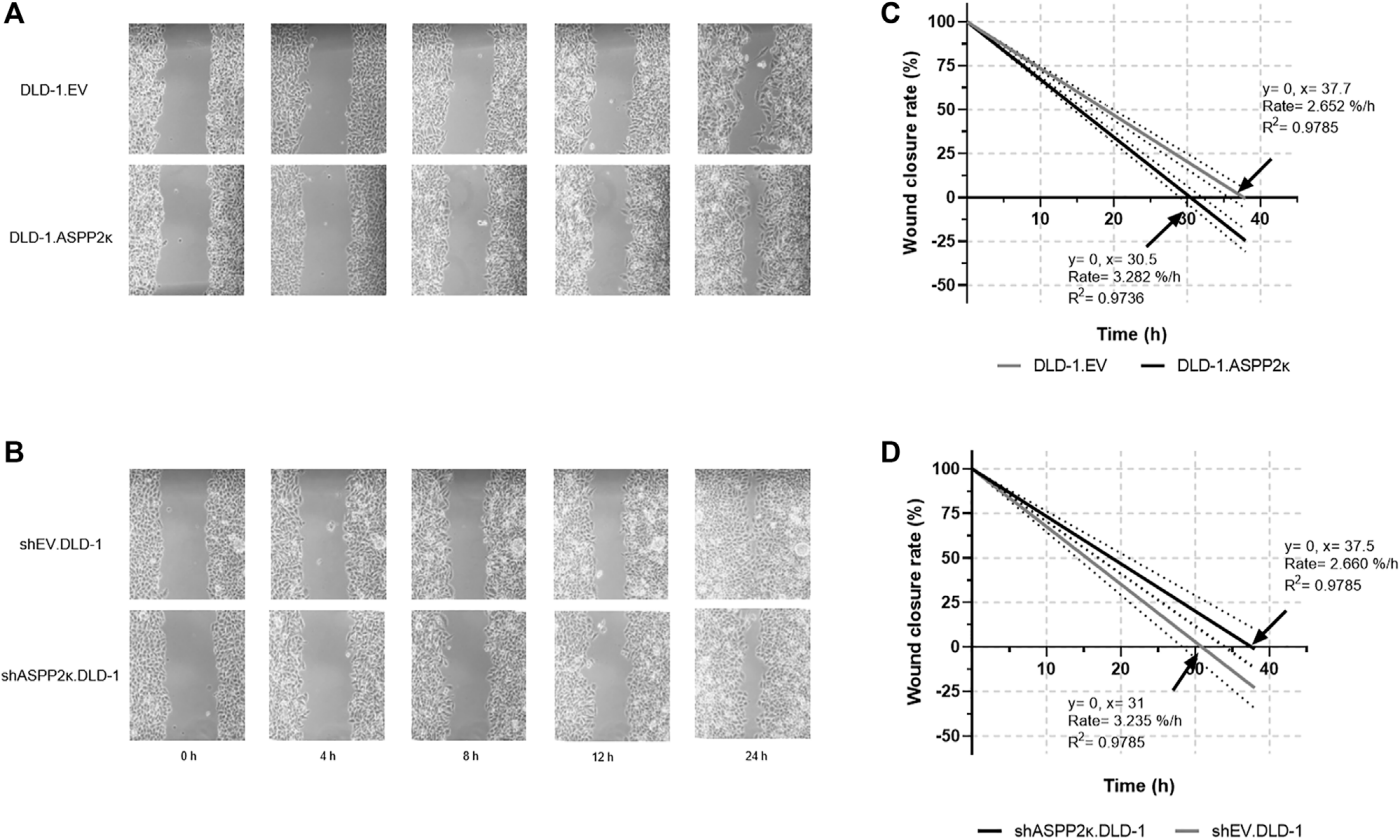
Exemplary wound healing experiments to visualize migratory behaviour of DLD-1.ASPP2κ **(A)** or shASPP2κ.DLD-1 **(B)** cells are shown. **(C,D)** Quantification of migration capacity as determined by wound closure rates of DLD-1.ASPP2κ **(C)** or shASPP2κ.DLD-1 **(D)** cell strains. EV, empty vector.

Vice versa, ASPP2κ-interference resulted in attenuated cellular migration in both CRC cell lines when compared to the respective EV controls. Specifically, DLD-1 cells showed 37% less cellular migration capacity in the transwell migration assay compared to their isogenic EV controls (Figure 5B). In HCT116 cells, ASPP2κ-interference led to a 30% reduction of migrating cells compared to the EV control (Figure 5D). To further confirm our findings that ASPP2κ enhanced CRC migration, we used a wound healing assay for the DLD-1 cell line (Figure 6). As expected, DLD-1 ASPP2κ overexpressing cells demonstrated enhanced cellular motility—resulting in an increased wound closure rate when compared to the DLD-1.EV control strains (∼3.3%/h vs 2.7%/h), (Figures 6A,C). In contrast, ASPP2κ -interference resulted in decreased cellular motility rates, which led to a delayed wound closure time of 37.5 h in comparison to the corresponding EV control strain wound closure time of 31 h (Figures 6B,D).

## Discussion

Although recent advances in the understanding, detection and treatment of CRC have improved outcomes for patients, CRC is still a major source of morbidity and mortality worldwide (Bray et al., 2018).

While early tumor stages can be treated curatively with surgery and chemotherapy, distant metastatic disease remains incurable except in selected circumstances (Chen et al., 2021). Therefore, understanding the cellular and molecular complexity of the disease remains crucial in order to unveil the molecular basis of CRC-tumorigenesis and to design new and more effective therapies (Ganesh et al., 2019) (Dienstmann et al., 2017).

Recent advances have shown the importance of identifying prognostic and predictive biomarkers in CRC as evidenced by several molecularly stratified treatment options. For example, the importance of microsatellite instability and TMB is evident in the use of immune-checkpoint inhibitors for specific CRC patient cohorts. Likewise, mutational status of RAS, BRAF, and ERBB2, as well as tumor-sidedness, are clinically relevant to guide therapy options (Sveen et al., 2020).

ASPP2 is a tumor suppressor that enhances apoptosis and promotes tumorigenesis via p53-dependent (Samuels-Lev et al., 2001) and p53-independent pathways (Kampa et al., 2009b; Wang et al., 2013). A role of dysfunctional ASPP2 in human neoplasms has been suggested by the observation that ASPP2 is downregulated in several tumor types, including human acute leukemia (Schittenhelm et al., 2013), choriocarcinoma (Mak et al., 2013), pancreatic cancer (Song et al., 2015) and diffuse large B-cell lymphoma (Lossos et al., 2002). In these studies, attenuated ASPP2 expression levels were associated with metastasis and poor clinical outcome (Lossos et al., 2002; Mak et al., 2013; Schittenhelm et al., 2013; Song et al., 2015). However, the precise mechanisms of ASPP2 regulation and function in these different contexts remain to be clarified. Further, different ASPP2 isoforms have been described, which have a profound functional impact on tumorigenesis and therapy resistance (Van Hook et al., 2017) (Schittenhelm et al., 2019).

In this context, we recently identified an exon-skipping splicing variant of ASPP2, named ASPP2κ, a C-terminally truncated isoform lacking the p53 binding sites, and defective in promoting stress-induced dependent apoptosis (Schittenhelm et al., 2019). In this report, we measure ASPP2κ expression in colorectal tumors obtained from patients and importantly find that ASPP2κ is overexpressed in tumor tissue compared to adjacent tumor-free tissue (Figure 1). In this small dataset, higher *ASPP2κ* expression levels were associated with clinical risk features—as each of the high expressors displayed at least one risk factor (such as tumor location, higher tumor infiltration stages, nodular involvement, distant metastasis or adverse mutations (Supplementary Table S1). However, the restricted number of patients clearly prevents a more profound analysis—and this topic has to be addressed in future studies using well-defined patient cohorts.

Intriguingly, we also found significant variation in ASPP2κ expression levels (Figures 1A,B). This suggests that additional pathways may be important in modulating ASPP2κ function. Consistent with a functional role in colorectal cancer therapeutics, we found that ASPP2κ expression inhibited oxaliplatin-induced apoptosis (Figure 3). Given that ASPP2κ is missing the C-terminal domain responsible for binding p53, this suggests its function may be involved in the p53-mediated pathway (Kampa et al., 2009b). Indeed, both CRC cell lines used harbored a TP53 WT background. Still, given the molecular heterogeneity in CRC (Sveen et al., 2020), including RAS/BRAF mutations, and that ASPP2 has other binding partners such as RAS (Wang et al., 2013), the precise mechanisms of ASPP2κ function remain to be elucidated. Nevertheless, our finding that ASPP2κ expression has functional consequences that mediate CRC tumor response to the key chemotherapy agent oxaliplatin opens important new avenues for investigation.

Previous studies have suggested that ASPP2 has other functions in addition to modulating apoptosis (Helps et al., 1995; Naumovski and Cleary, 1996; Chen et al., 2003; Meek et al., 2004; Kobayashi et al., 2005; Takahashi et al., 2005; Hakuno et al., 2007; Kampa et al., 2009a; Kampa et al., 2009b; Uhlmann-Schiffler et al., 2009; Wang et al., 2013). Using ASPP2κ enforced expression and ASPP2κ silencing in CRC cell lines, we found that ASPP2κ promotes tumor cell proliferation and migration. These tantalizing findings argue that ASPP2κ has additional functions beyond apoptosis modulation and suggests an important role in CRC tumor development. Given the stepwise development of colorectal tumors from polyp to invasive cancer (Fearon and Vogelstein, 1990), it will be interesting to interrogate ASPP2κ expression in pre-cancerous or early-stage lesions, which may reveal important mechanism in CRC oncogenesis that may have clinical therapeutic or early detection implications.

A potential role for the ASPP family in CRC has been suggested in previous studies (Yin et al., 2018). However, these studies did not distinguish between different full-length *ASPP2* and other isoforms (such as *deltaN-ASPP2* (Van Hook et al., 2017) or *ASPP2κ* (Schittenhelm et al., 2019))*.* This would be important because these different (and potentially functional counteracting) isoforms will make it difficult to establish robust clinical correlations. Our data strongly suggests that future studies will need to take the presence of different isoforms into account to understand the larger complex role of ASPP2 in colorectal cancer.

In summary, our findings demonstrate that ASPP2κ is expressed in human CRC tumors and that this may play a role in resistance to oxaliplatin-based chemotherapy. Moreover, our data also demonstrates that ASPP2κ may play a role in colorectal tumor development as evidenced by its ability to promote tumor cell proliferation and migration.

We provide important information that sets the stage for a larger scale investigation into the role of ASPP2κ as a prognostic and predictive biomarker that may play a role in making a positive impact for patients with colorectal cancer.

## Data Availability

The original contributions presented in the study are included in the article/Supplementary Material, further inquiries can be directed to the corresponding author.

## Glossary

18s: 18 S ribosomal RNA
7-AAD: 7-Aminoactinomycin
ANOVA: Analysis of Variance
APC: Allophycocyanin
ASPP1: Apoptosis Stimulating Protein of p53 protein 1
ASPP2: Apoptosis Stimulating Protein of p53 protein 2
Bbp: Bcl2-binding protein
cDNA: complementary DNA
CMV: Cytomegalovirus
Cp: Crossing point
CRC: Colorectal carcinoma
DMEM: Dulbecco′s Modified Eagle′s Medium
EV: Empty Vector
FACS: Fluorescence Activated Cell Sorting
FBS: Fetal Bovine Serum
GFP: Green Fluorescent Protein
iASPP: Inhibitor of Apoptosis Stimulating Protein of p53
IC50: Half Maximal Inhibitory Concentration
MEF: Mouse Embryonic Fibroblast
mRNA: messenger ribonucleic acid
Ox: Oxaliplatin
PBS: Phosphate Buffer Saline
PI: Propidium Iodide
qRT-PCR: quantitative reverse transcription polymerase chain reaction
RT: Room Temperature
SH3: SRC Homology 3
shRNA: short hairpin ribonucleic acid
siRNA: silencing ribonucleic acid
SV40: Simian virus 40
TP53: Tumor Protein 53
TP53BP2: Tumor Protein P53 Binding Protein 2
WHO: World Health Organization
WT: Wild Type
